# Assessing Recent Trends in Lumpectomy and Concomitant Oncoplastic Procedures: A National Insurance Database Analysis

**DOI:** 10.1245/s10434-026-19782-z

**Published:** 2026-05-11

**Authors:** Virginia Bailey, Luke Juckett, Sydney Pessin, Naomi Ghahrai, Lynn T. Dengel, Scott T. Hollenbeck

**Affiliations:** 1 Florida State University College of Medicine, Tallahassee, FL USA; 2 Department of Plastic Surgery, University of Virginia, Charlottesville, VA USA; 3 Virginia Commonwealth University School of Medicine, Richmond, VA USA; 4 Department of Surgery (Surgical Oncology), University of Virginia, Charlottesville, VA USA

**Keywords:** Breast-conserving therapy, Oncoplastic breast surgery, Lumpectomy, Adjacent tissue rearrangement, Breast reduction mammaplasty, Temporal trends

## Abstract

**Background:**

Following the National Surgical Adjuvant Breast and Bowel Project (NSABP) B-06 trial, breast-conserving therapy (BCT) has increasingly replaced mastectomy for eligible patients. Concurrently, oncoplastic surgical techniques have evolved to address contour deformities associated with lumpectomy and adjuvant radiation. While utilization of BCT has been well described, national trends in oncoplastic reconstruction remain less well defined. This study evaluates temporal trends in post-lumpectomy reconstruction using a large national insurance database.

**Patients and Methods:**

The PearlDiver™ national claims database was queried for patients undergoing lumpectomy (Current Procedural Terminology (CPT) 19301) from 2010 to 2020. Patients were stratified into four cohorts: lumpectomy alone, lumpectomy with mastopexy (LM; CPT 19316), lumpectomy with reduction mammaplasty (LR; CPT 19318), and lumpectomy with adjacent tissue rearrangement (LATR; CPT 14301/14302/19366). Primary outcomes included annual procedural volumes and proportional trends. Associations with demographics, comorbidities, and insurance status were analyzed using Welch’s analysis of variance (ANOVA) and chi-squared testing.

**Results:**

Among 486,368 patients, 92.3% underwent lumpectomy alone, while 4.6% underwent LATR, 1.6% LR, and 1.0% LM. Mean age differed significantly across cohorts (*p* < 0.001), with LR patients being youngest. Overall lumpectomy volume increased 27% over the decade, while lumpectomy with reconstruction increased 249%, driven by a 250% increase in LATR and a 622% increase in LR. Comorbidity burden was highest in the lumpectomy-alone cohort, whereas obesity and tobacco use were more prevalent in LR patients. Insurance distribution differed among groups (*p* < 0.001).

**Conclusions:**

Oncoplastic reconstruction following lumpectomy has increased from 2010 to 2020, led by adjacent tissue rearrangement techniques. These trends reflect an expanding emphasis on aesthetic and quality-of-life outcomes in breast-conserving therapy.

The National Surgical Adjuvant Breast and Bowel Project (NSABP) B-06 trial and subsequent landmark studies demonstrated equivalent oncologic outcomes across radical mastectomy, total mastectomy, and lumpectomy with radiation, with no significant differences in disease-free, distant-disease-free, or overall survival among the treatment groups^1–4^ Additional studies have supported breast-conserving therapy (BCT), even demonstrating a potential for improved survival over mastectomy. This body of evidence led to an increased adoption of BCT in the USA and became the preferred treatment for eligible patients.^1–4^ Since 2013, there has been a significant increase in the rate of lumpectomy surgery performed in the USA with a concomitant decrease in mastectomy rate.^5^ This is attributed to increased multidisciplinary care, improved patient education regarding equivalent survival with BCT, and evolving surgical options.^5^ Oncoplastic breast surgery (OPS) has emerged as an approach that is designed to reduce postoperative deformities following lumpectomy by integrating tumor resection with reconstruction techniques (breast reduction, mastopexy, and adjacent tissue rearrangement).^4^ A study evaluating more than 15,000 breast cancer cases showed that patients with newly diagnosed unilateral early stage breast cancer are electing BCT.^6^ OPS procedures have been broadly categorized into two levels on the basis of the extent of tissue resection. Level I procedures involve volume displacement for resections with under 20% of breast tissue whereas level II volume procedures involve either volume displacement or volume replacement when 20–50% of tissue is removed.

The goal of OPS is to preserve and maintain breast shape and symmetry while also offering the advantages of reduced surgical burden, shorter operative time, improved breast sensation, and enhanced psychosocial outcomes.^3,4^ This approach may also allow for wider excisions with better margin control and reduced need for conversion mastectomy, especially in patients with a higher tumor-to-breast ratio.^7–10^ While lumpectomies with OPS have improved cosmetic outcomes, they carry the risk of possible re-excision, radiation, or conversion to mastectomy if the tumor margins are not completely excised.^11^ Alternatively, mastectomy offers complete removal of breast tissue, eliminates the need for future reoperation, and avoids radiation therapy; however, it carries a higher risk of breast sensation loss and functional impairment.^12–14^ Direct comparisons between oncoplastic lumpectomy and mastectomy show that mastectomy patients experience greater declines in breast satisfaction, psychosocial well-being, and sexual well-being, and are also less likely to return to baseline quality of life after surgery.^15,16^ Lastly, mastectomies are also associated with higher decision regret in patients who were eligible for breast conservation but chose mastectomy instead.^16^ While numerous studies have shown an increasing trend in breast conservation therapy across the USA, trends in oncoplastic surgery approaches have been less clear.^2^ This study aims to evaluate temporal trends and demographic patterns in post-lumpectomy reconstruction procedures from 2010 to 2020 using a large national insurance dataset, PearlDiver.

## Patients and Methods

Data were obtained from the Pearldiver™ national administrative claims database, including all patients who underwent breast-conserving surgery from 1 January 2010 through 31 December 2020. Patients were identified using Current Procedural Terminology (CPT) code 19301 (lumpectomy). Cases were stratified into four exclusive cohorts on the presence of concurrent oncoplastic or reconstructive procedures performed at the time of lumpectomy: (1) lumpectomy alone, (2) lumpectomy with mastopexy (CPT 19316), (3) lumpectomy with reduction mammaplasty (CPT 19318), and (4) lumpectomy with adjacent tissue rearrangement, defined by the concurrent use of CPT 14301, 14302, and/or 19366. CPT 14301 was used to identify adjacent tissue transfer for reconstruction of lumpectomy defects measuring 30.1–60.0 cm^2^, with CPT 14302 capturing each additional 30.0 cm^2^ of tissue rearrangement performed during the same operative encounter, while CPT 19366 represented breast reconstruction using alternative oncoplastic volume displacement techniques following partial mastectomy.

The primary outcomes were annual procedural volumes from 2010 to 2020 and proportional trends relative to all lumpectomies and to all oncoplastic breast reconstructive procedures. Secondary outcomes included demographic characteristics and medical comorbidities. Patient variables extracted included age and insurance status (commercial, Medicare, Medicaid, governmental, or unknown). Comorbidities analyzed included chronic kidney disease, coagulopathy, chronic obstructive pulmonary disease (COPD), coronary artery disease, diabetes mellitus, hypertension, obesity, corticosteroid use, and current tobacco use.

Continuous variables were reported as means with standard deviations, and categorical variables were reported as frequencies with percentages. Comparisons of continuous variables across groups were performed using Welch’s analysis of variance (ANOVA). Categorical variables were analyzed using chi-squared testing. Temporal trends were analyzed using year-over-year procedural counts. All statistical analyses were performed using R version 4.5.2 (2025-10-31). Statistical significance was defined as a two-sided *p*-value < 0.05.

## Results

A total of 486,368 patients underwent lumpectomy between 2010 and 2020. Of these, 451,053 (92.3%) underwent lumpectomy alone, while 22,359 (4.6%) underwent lumpectomy with adjacent tissue rearrangement, 8068 (1.6%) with lumpectomy with breast reduction, and 4888 (1.0%) lumpectomy with mastopexy. Within the lumpectomy with adjacent tissue rearrangement patient group, the frequency of CPT-14301 was 64%, CPT-19366 was 37%, and CPT-14302 was 15%. The mean age across groups ranged from 61.9 ± 12.0 years (lumpectomy alone), 60.4 ± 11.7 (lumpectomy with adjacent tissue rearrangement), and 59.0 ± 11.3 (lumpectomy with mastopexy) to 57.3 ± 10.2 years (lumpectomy with reduction). Welch’s ANOVA demonstrated a significant difference in mean patient age among the four procedure groups (*p* < 0.001). Patients who underwent lumpectomy with breast reduction were significantly younger than all other cohorts (*p* < 0.001).

**Fig. 1:**
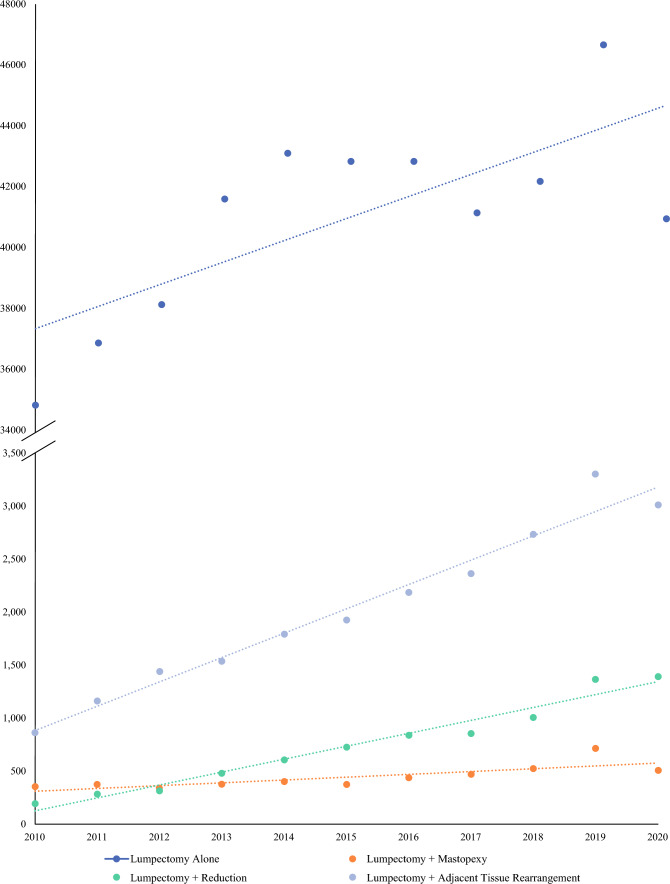
Temporal trends in lumpectomy and associated reconstruction procedures from 2010 to 2020; dotted lines indicate linear lines of best fit

Overall, the lumpectomy rate increased by 27% during the decade within this dataset (Fig. 1). Within this growth, lumpectomy-alone accounted for an 18% increase in procedural volume. The highest rate of increase was seen in lumpectomy with reconstructive procedures (249%); from 1410 cases in 2010 to 4922 cases in 2020. This increase in procedures was driven by a 622% increase in lumpectomy with breast reduction (193 cases in 2010 to 1395 cases in 2020), followed by a 250% increase in lumpectomy with adjacent tissue rearrangement procedures (863 cases in 2010 to 3017 cases in 2020), and a 44% in lumpectomy with mastopexy procedures (354 cases in 2010 to 510 cases in 2020). The largest absolute increase was in lumpectomy with adjacent tissue rearrangement procedures (863 cases in 2010 to 3017 in 2020). When annual trends were modeled using linear best-fit lines, lumpectomy alone demonstrated an increase of 735 cases a year, lumpectomy with adjacent tissue rearrangement had an increase of 230 cases annually, lumpectomy with reduction increased 122 cases a year, and lastly, lumpectomy with mastopexy increased 27 cases annually.

**Fig. 2:**
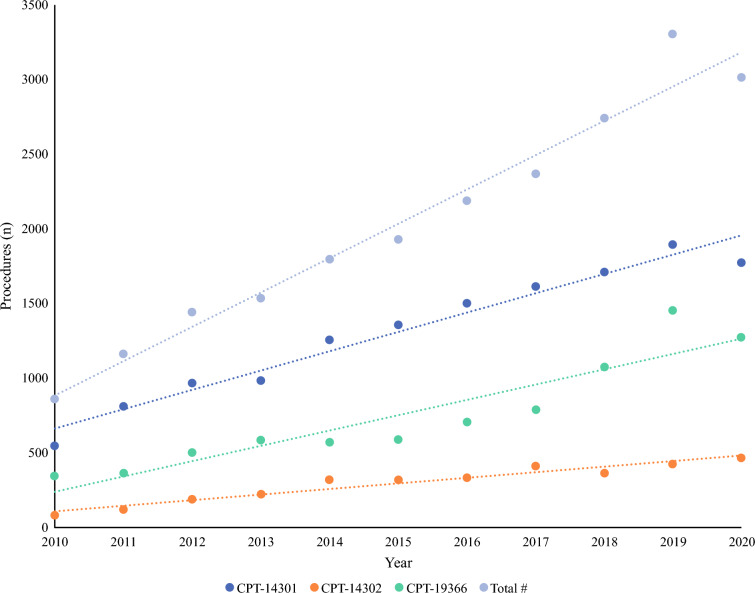
Temporal trends in lumpectomy with adjacent tissue rearrangement based on CPT code, including CPT 14301 (adjacent tissue transfer for defects 30.1–60.0 cm^2^), CPT 14302 (each additional 30.0 cm^2^ of adjacent tissue transfer), and CPT 19366 (breast reconstruction using alternative oncoplastic volume displacement techniques); dotted lines indicate linear lines of best fit

Three separate CPT codes contributed to the increase in total number of lumpectomies with adjacent tissue rearrangement performed. The 250% increase in lumpectomies with adjacent tissue rearrangement was driven by a 480% increase in CPT-14302 (810 cases in 2010 to 470 cases in 2020), a 270% increase in CTP-19366 (345 cases in 2010 to 1275 cases in 2020), and lastly a 224% increase of CPT-14301 (547 cases in 2010 to 1773 cases in 2020) (Fig. 2). When annual trends were modeled using linear best-fit lines, CPT-14301 increased by 130 cases per year, CPT-19366 increased by 103 cases per year, and lastly, CPT-14302 increased by 37 cases annually.

Among the surgical groups, there was a difference in prevalence of medical comorbidities amongst these surgical groups. As presented in Table 1, the prevalence of chronic kidney disease, coagulopathy, COPD, coronary artery disease, diabetes mellitus, hypertension, obesity, and tobacco use differed significantly among lumpectomy subgroups (*p* < 0.001). Hypertension (54.2%), diabetes mellitus (22.6%), coronary artery disease (11.3%), COPD (8.8%), chronic kidney disease (6.8%), and coagulopathy (4.5%), were highest in patients undergoing lumpectomy alone. Alternatively, obesity (35.5%), and tobacco use (21.3%) were more prevalent in patients undergoing lumpectomy with a breast reduction than the other surgical groups.

**Table 1:** Baseline comorbidities by lumpectomy and reconstruction subtype

	Lumpectomy alone	Lumpectomy + mastopexy	Lumpectomy + breast reduction	Lumpectomy + adjacent tissue rearrangement	*p*-Value
Chronic kidney disease	30,175 (7%)	200 (4%)	315 (4%)	1357 (6%)	< 0.05
Coagulopathy	20,434 (5%)	212 (4%)	359 (4%)	1148 (5%)	< 0.05
COPD	39,722 (9%)	359 (7%)	596 (7%)	1883 (8%)	< 0.05
Coronary artery disease	51,130 (11%)	419 (9%)	592 (7%)	2369 (11%)	< 0.05
Diabetes mellitus	101,846 (23%)	847 (17%)	1557 (19%)	5094 (23%)	< 0.05
Hypertension	244,375 (54%)	2220 (45%)	4078 (51%)	11,756 (53%)	< 0.05
Obesity	94,584 (21%)	969 (20%)	2851 (35%)	5517 (25%)	< 0.05
Steroid use	5265 (1%)	58 (1%)	111 (1%)	255 (1%)	0.37
Tobacco	82,390 (18%)	815 (17%)	1721 (21%)	4010 (18%)	< 0.05

**Fig. 3:**
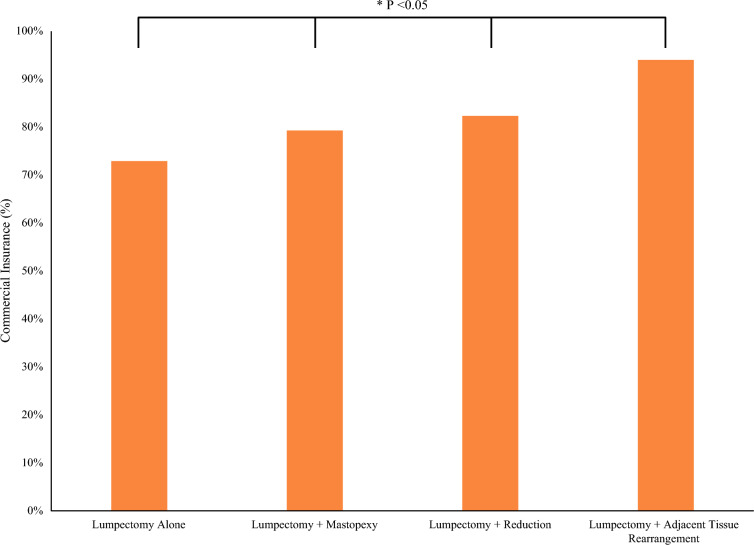
Percentage of patients with commercial insurance by lumpectomy subtype; the highest rate of commercial insurance was observed in patients undergoing lumpectomy with adjacent tissue rearrangement (94%), compared with lumpectomy alone (73%), lumpectomy with mastopexy (79%), and lumpectomy with reduction (82%); differences were statistically significant by chi-squared analysis (*p* < 0.05)

Finally, we sought to assess the variability of insurance types across the lumpectomy cohorts. As presented in Table 2, the type of insurance (commercial, governmental, Medicare, Medicaid) differed significantly between the lumpectomy subgroups (*p* < 0.001). The prevalence of commercial (85.1%), Medicaid (4.5%), and governmental (1.3%), were highest in patients undergoing lumpectomy with mastopexy. Medicare (21.7%) had the highest prevalence in patients undergoing lumpectomy alone. Among patients undergoing lumpectomy alone, the majority were commercially insured (72%), followed by Medicare (22%), Medicaid (4%), and governmental insurance (1%). In patients undergoing lumpectomy with mastopexy, lumpectomy with breast reduction, and lumpectomy with adjacent tissue rearrangement, patient insurance followed the same trend; the majority were commercially insured (81%; 85%; 76%), followed by Medicare (15%; 13%; 19%), Medicaid (4%; 4%; 4%), and governmental (1%, 1%, 1%), respectively. The proportion of patients with commercial insurance increased progressively across surgical complexity, with the highest rate observed in the lumpectomy with adjacent tissue rearrangement group (94%). Compared with all other lumpectomy subtypes, this difference was statistically significant by chi-squared analysis (*p* < 0.05) (Fig. 3).

**Table 2:** Insurance payer distribution by lumpectomy and reconstruction subtype

	Commercial	Governmental (CMS)	*p*-Value
Lumpectomy alone	324,895 (73%)	121,282 (27%)	< 0.05
Lumpectomy + mastopexy	3961 (79%)	1037 (21%)	< 0.05
Lumpectomy + reduction	6869 (82%)	1476 (18%)	< 0.05
Lumpectomy + adjacent tissue rearrangement	17,056 (94%)	1107 (6%)	< 0.05

## Discussion

With the oncologic safety of breast conservation firmly established by NSABP B-06, attention in breast surgery has increasingly turned toward reconstructive strategies that enhance cosmetic and quality-of-life outcomes.^1–4^ This pivotal body of evidence fundamentally reshaped surgical management of breast cancer and drove the widespread adoption of breast-conserving therapy as the preferred approach for appropriately selected patients. Shortly after the dissemination of this information, trends in population-level breast surgery shifted.^3^ A National Surgical Quality Improvement Program (NSQIP), Surveillance Epidemiology and End Results (SEER), and National Cancer Database (NCDB) study shows that mastectomy rates have decreased significantly after 2013, with a parallel increase of lumpectomy rates across the USA.^5^ BCT became the preferred and gold standard approach to oncological breast treatment. Furthermore, physicians sought improved surgical technique to optimize cosmoses and quality of life outcomes.

Patient experience is emerging to be a major factor in the increase in oncoplastic reconstruction, as there is similar cosmetic satisfaction between breast-conserving therapy and mastectomy with reconstruction.^17,18^ It has been shown that radiation and reconstruction type significantly impact cosmetic outcomes.^11–14,18–22^ Multiple studies using validated patient-reported outcome measures (such as the BREAST-Q) consistently show that breast-conserving approaches, especially when oncoplastic techniques are used, result in better patient-reported satisfaction and well-being than mastectomy.^16,19–23^ For example, a large prospective cohort (ANTHEM study) found that women who underwent oncoplastic breast-conserving surgery reported significant improvements in satisfaction with breasts and psychosocial well-being at both 3 and 12 months postoperatively.^11^ Similarly, a systematic review and meta-analysis found that oncoplastic surgery outperformed standard partial mastectomy in all BREAST-Q domains, and breast-conserving surgery (with or without oncoplastic techniques) was associated with higher satisfaction and quality of life than mastectomy.^14,24^ As patient-reported outcomes continue to become essential in shared decision-making between patient and physician, lumpectomy with oncoplastic reconstruction provides a balanced option that combines oncologic safety with aesthetic optimization.^17,18^

Oncoplastic techniques have increased over the last decade to optimize cosmetic outcomes, as traditional lumpectomies leave significant contour deformities in up to 30% of patients.^4^ It is thought that oncoplastic techniques, such as breast reduction, mastopexy, and adjacent tissue rearrangement following lumpectomy improve aesthetic outcomes by addressing volume displacement and asymmetry.^4,5,7,25–29^ Recent literature further supports the growing role of these procedures, demonstrating that oncoplastic breast-conserving surgery is associated with lower re-excision rates, similar oncologic outcomes, and improved cosmetic satisfaction compared with standard breast-conserving surgery, even in the setting of neoadjuvant systemic therapy.^30,31^ Additional evidence suggests that specific techniques such as bilateral reduction mammoplasty may provide wider surgical margins, improved breast symmetry, and more effective radiotherapy planning without increasing local recurrence or compromising survival.^32^ More broadly, oncoplastic surgery is increasingly being integrated into multidisciplinary breast cancer care because it improves breast contour, symmetry, and patient satisfaction while maintaining oncologic safety.^33^ These factors likely explain the rapid expansion of lumpectomy with adjacent tissue rearrangement, breast reduction, and mastopexy in our database findings.

Furthermore, there is a growing emphasis on esthetic outcomes and oncoplastic techniques being incorporated into breast surgical oncology fellowship training. The increase in adjacent tissue rearrangement may be partially explained by this increased awareness. While this study did not attempt to determine whether these procedures were being performed by breast surgeons or plastic surgeons, it is possible that the more basic rearrangement techniques are becoming more commonly performed by breast surgeons as a standard aspect of addressing a lumpectomy defect at the time of wound closure.^34,35^

Comorbidity patterns likely played a substantial role in determining reconstructive approach. The highest rates of hypertension, diabetes, coronary artery disease, COPD, chronic kidney disease, and coagulopathy occurred in patients receiving lumpectomy alone, which aligns with a risk-averse surgical strategy in older or medically fragile patients where prolonged operative time or additional tissue manipulation may increase complication risk. In contrast, obesity and tobacco use were more prevalent among the breast reduction cohort, both factors associated with symptomatic macromastia and a younger demographic profile. Breast reductions are especially beneficial in large-breasted patients, improving resection and symmetry.^4,5^ Younger patients may place increased prioritization on esthetic outcomes and are more physiologically able to undergo more invasive and bilateral surgical approaches to achieve this result. Our data align with this principle. Prior studies have noted that oncoplastic reduction is frequently selected in patients seeking both oncologic tissue removal and functional improvement, which may explain this trend.^4,5^ These patterns suggest that patient comorbidity burden may be associated with oncoplastic eligibility and utilization.

Insurance status also appears to serve as a structural determinant of access to oncoplastic reconstruction. Prior studies have demonstrated that patients with public insurance (Medicaid or Medicare) or no insurance undergo oncoplastic breast surgery and post-lumpectomy reconstruction at significantly lower rates compared with privately insured patients, even after adjusting for clinical and demographic factors.^26,36^ Socioeconomic status, race, and geographic location further compound these disparities, reinforcing insurance coverage as a key barrier to equitable access.^37–39^ Consistent with these findings, our data identify that lumpectomy with OPS is not accessed equally, as commercial insurance status may facilitate reconstructive access. Medicare and Medicaid create practical limitations though reimbursement models, referral access, and institutional availability to perform lumpectomy with OPS. These data are limited in that insurance status is likely impacted by age, previously shown to be a factor in post-lumpectomy reconstruction implementation. This may be due to perceived higher operative risk by surgeons, less emphasis on esthetic outcomes among older patient populations, and more conservative reimbursement environments in Medicare. This information mirrors documented disparities in reconstructive access nationally.^17,18^

This study assessed a large population over 10 years to capture real-world patterns in oncologic breast surgery and reconstructive trends. The granular CPT-level procedural resolution allows for the study of nationwide trends in this population. However, this dataset provides no tumor-specific details about stage or breast volume ratios and does not provide any postoperative outcomes. Future research should include patient-reported outcomes between the various oncoplastic reconstructive options, cost analysis for long-term stability of these trends, and postoperative outcomes between these patient groups.

## Conclusions

Lumpectomy with oncoplastic reconstruction (breast reduction, mastopexy, and adjacent tissue rearrangement) utilization has risen substantially from 2010 to 2020. This increase has been mainly driven by lumpectomy with adjacent tissue rearrangement and breast reduction procedures. These findings reflect the evolving philosophy of breast-conserving therapy, where esthetic preservation is now integral to cancer care. As oncoplastic reconstruction becomes increasingly integrated into standard breast-conserving therapy, future research should prioritize the intersection of insurance status, access to care, and patient-reported outcomes to better define and address disparities in reconstructive utilization.
