# Correction: Assessing Recent Trends in Lumpectomy and Concomitant Oncoplastic Procedures: A National Insurance Database Analysis

**DOI:** 10.1245/s10434-026-19857-x

**Published:** 2026-05-19

**Authors:** Virginia Bailey, Luke Juckett, Sydney Pessin, Naomi Ghahrai, Lynn T. Dengel, Scott T. Hollenbeck

**Affiliations:** 1 Florida State University College of Medicine, Tallahassee, FL USA; 2 Department of Plastic Surgery, University of Virginia, Charlottesville, VA USA; 3 Virginia Commonwealth University School of Medicine, Richmond, VA USA; 4 Department of Surgery (Surgical Oncology), University of Virginia, Charlottesville, VA USA

**Correction to: Annals of Surgical Oncology** 10.1245/s10434-026-19782-z

In the original online version of this article Luke Juckett’s and Naomi Ghahrai’s family names were misspelled.

The original article was corrected.

